# Supplementary material for: The genome of the cryopelagic Antarctic bald notothen, Trematomus borchgrevinki
Source: G3 (Bethesda). 2024 Nov 16;15(1):jkae267. doi: 10.1093/g3journal/jkae267 (PMC11708224; doi:10.1093/g3journal/jkae267)
Supplement: jkae267_Supplementary_Data [file jkae267_supplementary_data.pdf]

Supplementary file:

The genome of the cryopelagic Antarctic bald notothen, *Trematomus borchgrevinki*

Niraj Rayamajhi[1], Angel G. Rivera-Colón[2], Bushra Fazal Minhas[3], C.-H. Christina Cheng[1], Julian M. Catchen[1*]

[1]Department of Evolution, Ecology, and Behavior, University of Illinois at Urbana-Champaign, Urbana, Illinois, USA

[2]Institute of Ecology and Evolution, University of Oregon, Eugene, Oregon, USA

[3]Informatics Program, University of Illinois at Urbana-Champaign, Urbana, Illinois, USA

*Corresponding Author: Julian Catchen (jcatchen@illinois.edu)

Figure S1: Hi-C contact map for the *Trematomus borchgrevinki* genome assembly

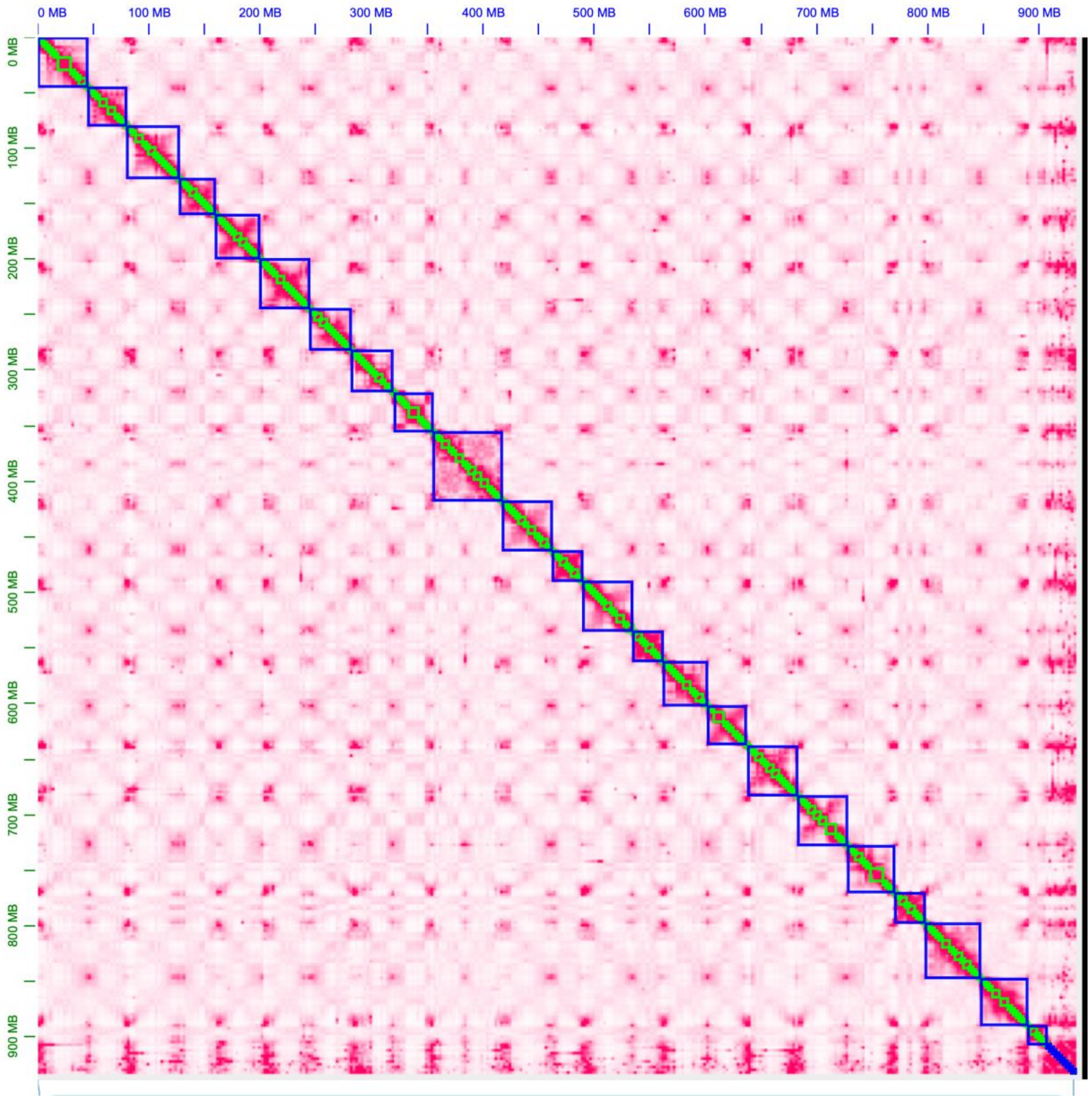

Heat map describing the interactions between Hi-C read pairs across the *T. borchgrevinki* assembly. The scaffolded assembly is composed of 2,094 scaffolds (blue boxes), each comprising one or more assembled contigs (green boxes). The 23 largest scaffolds (>1 Mbp) account for 97.6% of the assembly and represent the 23 chromosomes expected in the species. These 23 chromosome-scale scaffolds include one larger sequence product of a species-specific chromosomal fusion (the tenth sequence, starting from the top left corner). The Hi-C contact map figure was generated by `Juicebox` version 2.17.00 (Dudchenko et al., 2018; Durand et al., 2016).

Figure S2: Updated notothenioid phylogeny including *T. borchgrevinki*

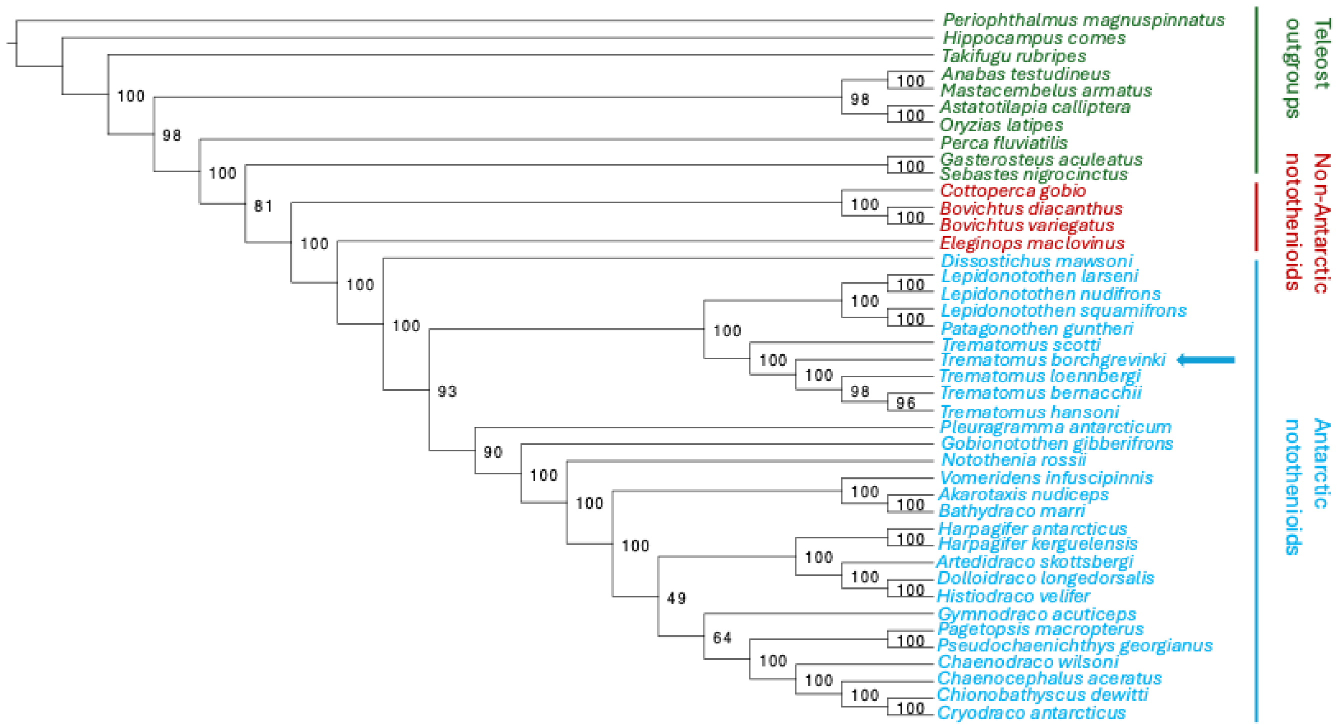

Midpoint-rooted maximum likelihood tree for 42 species, including 28 Antarctic notothenioids, four non-Antarctic notothenioids, and 10 teleost outgroups. The tree was generated by adding *T. borchgrevinki* BUSCO orthologs to the dataset generated by Bista *et al.*, (2023). Branch lengths describe the number of substitutions per amino-acid site. Ultra-fast bootstrap support values are shown for each non-outgroup node. Vertical colored bars on the right show the placement of Antarctic notothenioids (blue), non-Antarctic notothenioids (red), and teleost outgroups (green). The placement of *T. borchgrevinki* (highlighted by the arrow) within other *Trematomus* species is recovered with a bootstrap support of 100%.

Figure S3: Validation of the AFGP locus assembly in *T. borchgrevinki*

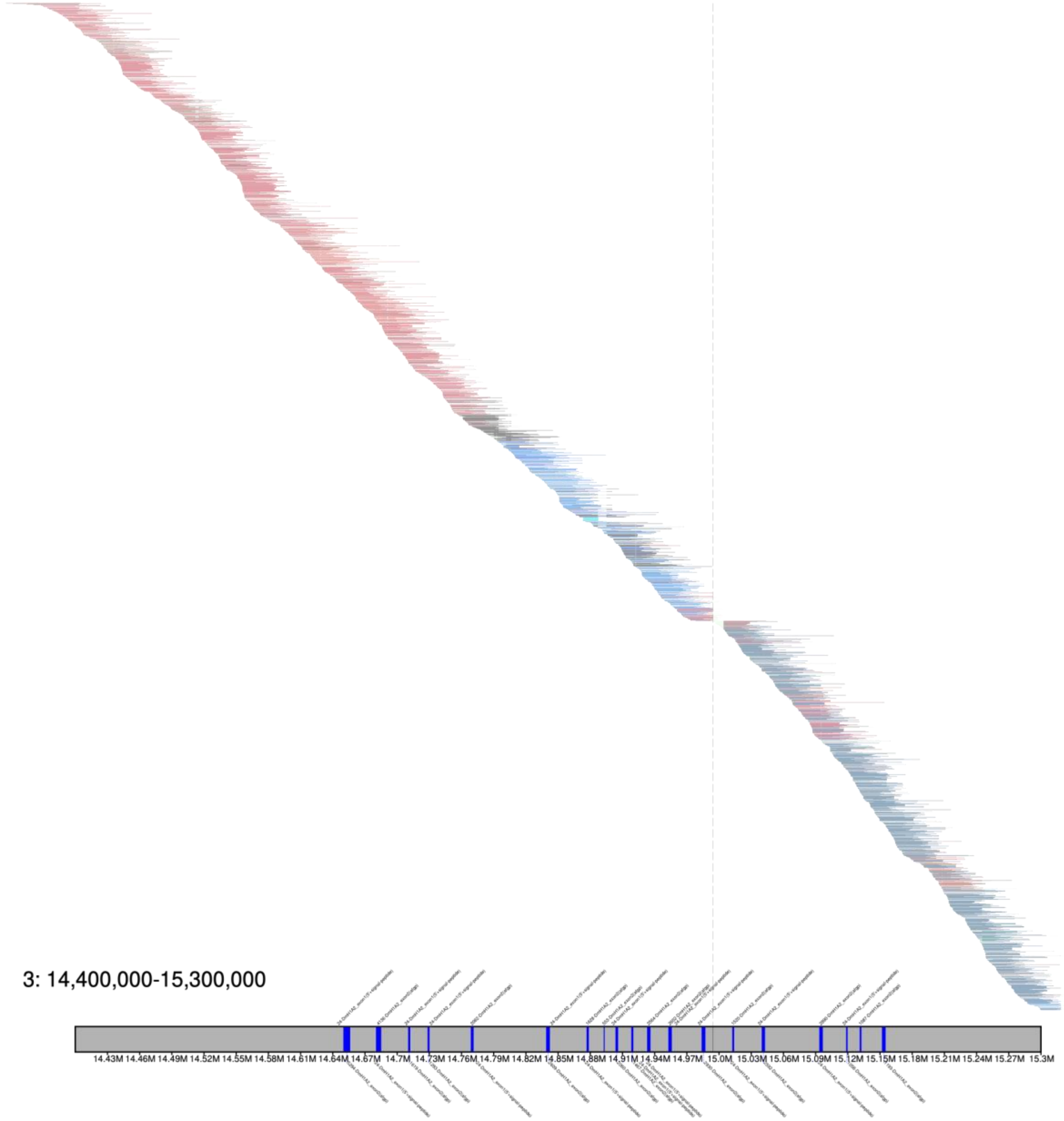

`Klumpy` (Madrigal et al., 2024) alignment plot showing the tiling of reads and AFGP k-mer klumps in the *T. borchgrevinki* AFGP locus. The plot shows alignments on chromosome 3, between the coordinates of 14.4-15.3 Mbp. The bottom horizontal bar represents chromosome 3 with the blue boxes indicating the location of AFGP exons. Horizontal blue and red lines denote the aligned reads, with colors denoting the groups assigned by `klumpy alignment_plot --group_seqs`. Except for a gap (shown by the vertical dashed line) at ~14.994 Mbp—which does not overlap any of the AFGP annotations, we observe a complete tilling of reads spanning the whole sequence, suggesting the proper assembly of the locus.

Table S1: Annotation of genes within the Antifreeze glycoprotein/Trypsinogen-like protease locus in chromosome 3

Locus genes	Start	End	Status	Gene features description details
HSL (lipe)	14,483,959	14,503,575	Complete, intact	
TRYP1_1	14,565,790	14,567,352	Complete, intact	
TRYP1-2	14,569,947	14,571,319	Complete, pseudogene	One premature stop each in exon-2 and exon-3; truncated 5' of exon-4
TRYP1_3	14,577,878	14,579,315	Complete, intact	
TRYP1_4	14,589,908	14,591,375	Complete, intact	
TRYP1_5	14,595,086	14,596,528	Complete, intact	
TRYP1_6	14,600,231	14,601,673	Complete, intact	
TRYP1_7	14,605,375	14,606,817	Complete, intact	
TRYP1_8	14,609,030	14,610,250	Complete, intact	
TRYP1_9	14,611,542	14,613,518	Complete, intact	
TRYP1_10	14,616,538	14,617,979	Complete, intact	
TRYP3_1	14,621,086	14,623,474	Complete, intact	
TLP_1	14,626,412	14,630,681	Incomplete, pseudogene	Missing exon-3 and truncated 5' in exon-4
Chimeric AFGP/TLP_1	14,645,735	14,659,517	Complete, pseudogene	Six frameshifts in exon-2's (Ala-Ala-Thr)$_n$ repeats
Tryp3_2	14,674,235	14,676,712	Complete, pseudogene	Premature stop codon in exon-2 and exon-5
Chimeric AFGP/TLP_2	14,678,552	14,687,919	Complete, pseudogene	Two frameshifts in (Ala-Ala-Thr)$_n$ repeats of exon-2
AFGP_1	14,708,553	14,712,196	Complete, intact	
AFGP_2	14,728,353	14,731,688	Complete, pseudogene	One frameshift in (Ala-Ala-Thr)$_n$ of exon-2
AFGP_3	14,768,626	14,773,218	Complete, intact	
AFGP_4	14,838,846	14,844,269	Complete, intact	
AFGP_5	14,874,725	14,878,639	Complete, intact	
AFGP_6	14,892,492	14,893,418	Incomplete, Pseudogene	Missing exon-1
AFGP_7	14,901,609	14,905,944	Complete, intact	
AFGP_8	14,918,141	14,922,040	Complete, intact	
AFGP_9	14,931,786	14,936,088	Complete, intact	
AFGP_10	14,952,744	14,957,584	Complete, intact	
AFGP_11	14,981,773	14,987,280	Complete, intact	
AFGP_12	15,010,386	15,014,077	Complete, intact	
AFGP_13	15,037,823	15,042,864	Complete, intact	
AFGP_14	15,091,856	15,096,715	Complete, intact	
AFGP_15	15,116,746	15,119,815	Complete, intact	
Chimeric AFGP/TLP_3	15,129,612	15,136,544	Complete, intact	
TRYP3_3	15,142,025	15,147,454	Complete, Pseudogene	Frameshift mutation at 3' end of exon-3
AFGP_16	15,149,687	15,155,255	Complete, intact	
TOMM40	15,205,539	15,210,987	Complete, intact	

References:

Bista, I., Wood, J. M. D., Desvignes, T., McCarthy, S. A., Matschiner, M., Ning, Z., Tracey, A., Torrance, J., Sims, Y., Chow, W., Smith, M., Oliver, K., Haggerty, L., Salzburger, W., Postlethwait, J. H., Howe, K., Clark, M. S., William Detrich, H., Christina Cheng, C.-H., … Durbin, R. (2023). Genomics of cold adaptations in the Antarctic notothenioid fish radiation. *Nature Communications*, *14*(1), 3412. https://doi.org/10.1038/s41467-023-38567-6

Dudchenko, O., Shamim, M. S., Batra, S. S., Durand, N. C., Musial, N. T., Mostofa, R., Pham, M., Glenn St Hilaire, B., Yao, W., Stamenova, E., Hoeger, M., Nyquist, S. K., Korchina, V., Pletch, K., Flanagan, J. P., Tomaszewicz, A., McAloose, D., Pérez Estrada, C., Novak, B. J., … Aiden, E. L. (2018). *The Juicebox Assembly Tools module facilitates* de novo *assembly of mammalian genomes with chromosome-length scaffolds for under $1000*. https://doi.org/10.1101/254797

Durand, N. C., Shamim, M. S., Machol, I., Rao, S. S. P., Huntley, M. H., Lander, E. S., & Aiden, E. L. (2016). Juicer Provides a One-Click System for Analyzing Loop-Resolution Hi-C Experiments. *Cell Systems*, *3*(1), 95–98. https://doi.org/10.1016/j.cels.2016.07.002

Madrigal, G., Minhas, B. F., & Catchen, J. (2024). Klumpy: A tool to evaluate the integrity of long-read genome assemblies and illusive sequence motifs. *Molecular Ecology Resources*, e13982. https://doi.org/10.1111/1755-0998.13982
